# Dual targeting of the antagonistic pathways mediated by Sirt1 and TXNIP as a putative approach to enhance the efficacy of anti-aging interventions

**DOI:** 10.18632/aging.100035

**Published:** 2009-03-31

**Authors:** Shaker A. Mousa, Christine Gallati, Tessa Simone, Emmy Dier, Murat Yalcin, Evgeny Dyskin, Sudha Thangirala, Christine Hanko, Abdelhadi Rebbaa

**Affiliations:** The Pharmaceutical Research Institute at Albany, Albany College of Pharmacy and Health Sciences, Rensselaer, NY 12144, USA

**Keywords:** Sirt1, TXNIP, DHEA, oxidative stress, metabolism, aging, stem cells

## Abstract

The organism's ability to regulate oxidative stress and metabolism is well recognized as a major determinant of longevity. While much research interest in this area is directed towards the study of genes that inhibit oxidative stress and/or improve metabolism, contribution to the aging process of genes with antagonistic effects on these two pathways is still less understood. The present study investigated the respective roles of the histone deacetylase Sirt1 and the thioredoxin binding protein TXNIP, two genes with opposite effects on oxidative stress and metabolism, in mediating the action of putative anti-aging interventions. Experiments were carried out *in vitro* and *in vivo* to determine the effect of proven, limited calorie availability, and unproven, resveratrol and dehydroepiandrosterone (DHEA), on the expression of Sirt1 and TXNIP. The results indicated that limited calorie availability consistently inhibited TXNIP in cancer and in normal cells including stem cells, however, it only slightly induced Sirt1expression in cancer cells. In contrast, resveratrol had a biphasic effect, and DHEA inhibited the expression of these two genes in a tissue specific manner, both *in vitro* and *in vivo*. Whereas all the three approaches tested inhibited TXNIP through the glycolytic pathway, DHEA acted by inhibiting G6PD and resveratrol through the activation of AMPK. In light of previous reports that Sirt1 induces AMPK-mediated signaling pathway, our findings point to the possibility of a negative relationship between Sirt1 and TXNIP that, if validated, can be exploited to improve the efficacy of putative anti-aging interventions.

## Introduction

Manipulation of metabolism and resistance to oxidative stress has been shown to promote longevity of small organisms [1,2], and to some extent, these same mechanisms appear to act also in mammals despite considerable divergence during evolution [1]. One of the most described genes that inhibit oxidative stress and improve glucose metabolism is the histone deacetylase Sirt1 (for Silent information regulator T1). Evidence has been provided recently that Sirt1 negatively regulates oxidative stress [3-5] and protects cells against  damage induced by H_2_O_2_, UV radiation, chemicals, and high caloric intake [6,7]. In addition, this gene was found to regulate cellular metabolism through the stimulation of glucose uptake [8,9] and insulin secretion [10-12], making it a promising target of putative anti-aging interventions [6,10,13].

Little is known however about the contribution to the aging process of genes that act, in an opposite manner to Sirt1, to induce oxidative stress and disrupt glucose metabolism.  An example of such genes is the thioredoxin interacting protein (TXNIP) [14-16]. Early reports have demonstrated that TXNIP enhances cellular susceptibility to oxidative stress through direct interaction with the anti-stress enzyme, thioredoxin, and inhibition of its detoxifying functions [17]. Over-expression of this gene was postulated to cause the accumulation of reactive oxygen species and apoptotic cell death [18,19]. More recently, evidence was provided that TXNIP may also act as a mediator of cellular metabolism. For instance, this gene was found to mediate glucose-induced apoptotic death in pancreatic beta cells, suggesting a causative relationship between TXNIP and type 2 diabetes [20,21]. In support of this concept, TXNIP deficiency improved glucose uptake and attenuated diabetic symptoms in animal models [16,22,23]. Also, and as might be expected from these findings, an inverse correlation between TXNIP expression and longevity was recently reported [24-26] supporting the notion that this gene may act to negatively regulate the aging process.

**Figure 1.**
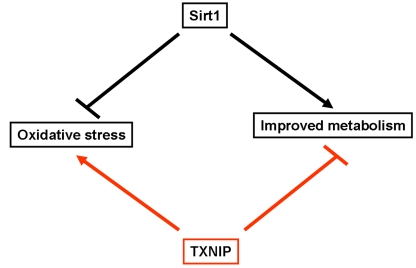
Schematic representation highlighting the opposite effects of Sirt1 and TXNIP on oxidative stress and metabolism. Sirt1 has been shown to inhibit oxidative stress and improves glucose uptake as well as insulin secretion. In contrast, TXNIP was found to enhance cellular susceptibility to oxidative stress and inhibit glucose uptake and insulin secretion.

Taking into account this antagonistic relationship between Sirt1 and TXNIP regarding their effects on oxidative stress and metabolism (Figure 1), it is important to determine if, and how putative anti-aging approaches would affect the function of these two genes, and whether a regulatory relationship might exist between them. To address these possibilities, the present study investigated the effects of limited glucose availability (used here to mimic the effects of calorie restriction), the Sirt1 activator (resveratrol), and the hormone dehydroepiandrosterone (DHEA), on expression of Sirt1 and TXNIP in different cellular systems, including cancer, stem cells and a mice model. A potential link between these two genes and the underlying mechanisms leading to their regulation were also investigated. Our results indicated that each of the treatment approaches tested exerted effects on the expression of Sirt1 and TXNIP, however to various degrees. Interestingly limited glucose availability was the only approach that consistently reduced TXNIP expression in all the systems studied. Resveratrol and DHEA exerted inhibitory effects on TXNIP in a tissue specific manner, in addition, they were found to act on separate branches of the glycolytic pathway mediated respectively by AMPK and Glucose 6 phosphate dehydrogenase respectively. Overall, our findings suggest that dual targeting of antagonistic pathways implicated in the aging process must be considered in the design of anti-aging strategies. They also shed light on TXNIP as a downstream target of Sirt1 and therefore as a potential marker to predict or to evaluate the efficacy of putative anti-aging therapeutics.

**Figure 2.**
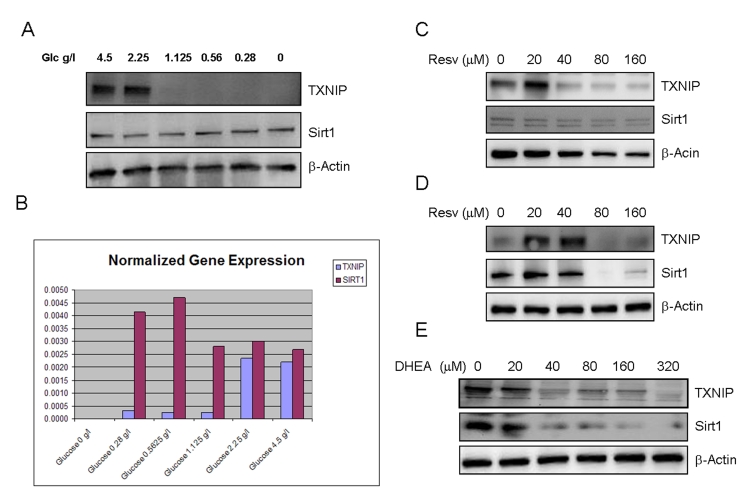
Effects of limited glucose availability, resveratrol and DHEA on expression of TXNIP and Sirt1 in cancer cells. Panel **A**. SaOS2 cells were incubated in the presence of reduced glucose levels in the culture medium for 48 hours. Expression of TXNIP and Sirt1 were determined by Western blot using specific antibodies. Antibody to β-actin was used as a loading control. Panel **B**. Respective expression of TXNIP and Sirt1 in response to limited glucose availability, measured by quantitative real time PCR (Q-PCR). SaOS2 cells were incubated in the presence of the indicated amounts of glucose for 48 hours, QPCR was then performed to detect expression of TXNIP and Sirt1 using specific primers. Panel **C** and **D**, the RGC (panel **C**) and SaOS2 (panel **D**) cells were subjected to treatment with increasing concentrations of resveratrol (Resv.) for 48 hours followed by Western blot as described in Panel **A**. Panel **E**, SaOS2 cells were treated with increasing amounts of DHEA and probed for expression of Sirt1 and TXNIP, β-actin was used as a loading control.

## Results

### Respective effects of limited glucose availability, resveratrol and DHEA on expression of Sirt1 and TXNIP in cancer cells

The effects of limited glucose availability (used here to mimic the effect of calorie restriction), resveratrol (an activator of Sirt1) and DHEA (used for hormone replacement therapy), on the expression of TXNIP and Sirt1 were determined by Western blot using specific antibodies to the corresponding proteins (Figure 2).  The data shows that cellular incubation with decreasing concentrations of glucose resulted in a dramatic loss of TXNIP expression (Figure 2A). Similar results were observed by using other cancer cell lines (Supplemental Data 1). Limited glucose availability was also found to cause a slight increase in the expression of Sirt1 (Figure 2A),  supporting  the  notion  that this  gene  acts as  a mediator of caloric restriction [27]. PCR analysis (Figure 2B) confirmed the previous findings and suggests that altered expression of TXNIP and Sirt1 in response to glucose deprivation occurred at the transcriptional level.

Curiously, resveratrol had a biphasic effect on the expression of TXNIP, with a stimulatory action at low concentrations and inhibition at higher ones (Figure 2C and 2D). Even more intriguing is the finding that resveratrol suppressed the expression of its own target, Sirt1, in both RGC cells (Figure 2C) and SaOS2 cells (Figure 2D), at the same concentrations that inhibit TXNIP. These data suggest that regardless of the concentration used, resveratrol may exert unwanted effects. At low concentrations, it induces the expression of TXNIP, a potential antagonist with regards to Sirt1 effect oxidative stress and metabolism, and at high concentrations, it inhibits expression of its own target. Based on this, the dosage at which resveratrol is administered should be carefully determined in order to achieving beneficial effects of this molecule.

Dehydroepiandrosterone, DHEA is a hormone thought to play a role in aging based on the observation that its levels decline dramatically with age to reach values of 30 to 20 % at 70 ~80 years [28]. Although some studies have suggested that replacement of this hormone at later age may have beneficial effects on illnesses such as atherosclerosis [29], autoimmune diseases [30], obesity [31], and neurodegeneration [32], many studies failed to demonstrate any beneficial effect of this hormone on aging. At the molecular level, DHEA was shown to improve glucose uptake [33] and reduce the formation of reactive oxygen species [34], however, the putative roles of Sirt1 or TXNIP in mediating these actions are not known. Here we show that DHEA acts as a dual regulator of these two genes (Figure 2E). Exposure to increasing concentrations of this hormone resulted in decreased expression of both TXNIP and Sirt1. While the inhibition of TXNIP by this hormone is desirable, that of Sirt1 is not, suggesting that the anti-aging effects of DHEA, if any, would be hindered by its inhibitory effect on Sirt1 and related pathways.

Overall, the findings presented in Figure 2 indicate that reduced calorie intake would rank the best among three approaches tested in this system. In contrast, suppression of Sirt1 by both resveratrol and DHEA may represent a major limitation to their potential use as anti-aging therapeutics. Nevertheless, a proper understanding of how these two treatments inhibit Sirt1 will help in the development of approaches to avoid this "side effect" and improve their efficacy.

### Expression of Sirt1 and TXNIP in normal cells

**Figure 3.**
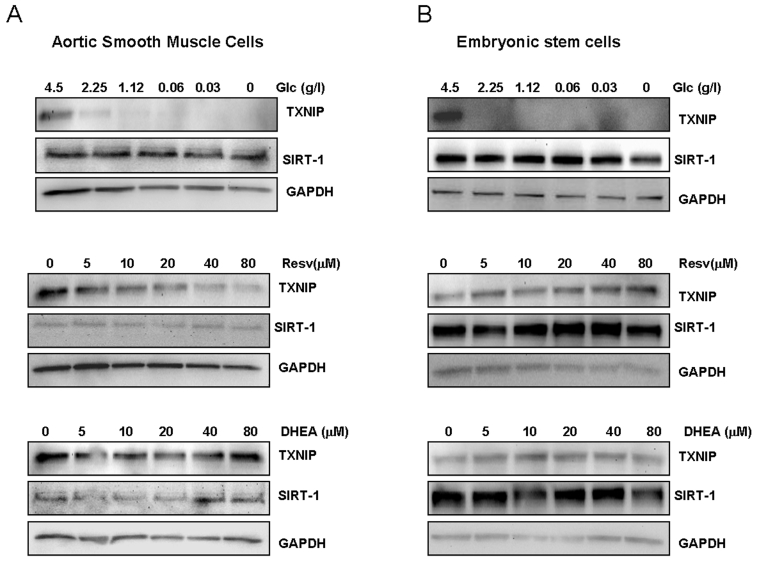
Effects of limited glucose availability, resveratrol and DHEA on expression of TXNIP and Sirt1 in normal cells. Aortic smooth muscle cells (panel **A**) and human embryonic stem cells (panel **B**) were treated as described in Figure 1 for cancer cells. Expression Sirt1 and TXNIP were analyzed by Western blot using specific antibodies. Antibody to GAPDH was used as loading control. (Glc: glucose., Resv: Resveratrol).

In order to obtain information on the physiological relevance of targeting Sirt1 and TXNIP in normal tissues, we investigated the effect of limited glucose availability, resveratrol and DHEA in differentiated aortic smooth muscle cells, and non-differentiated, human embryonic stem cells. As shown in Figure 3A, limited glucose availability inhibited TXNIP expression in smooth muscle cells, without any significant effect on Sirt1. Similar effects were also observed with resveratrol which inhibited the expression of TXNIP in a dose dependent manner. DHEA was without effect on any of the two genes (Figure 3A), raising the possibility that the glycolytic pathway targeted by this hormone may be altered in normal versus cancer cells. Of note, no significant changes in Sirt1 expression was observed in cells treated with any of the approaches tested, further confirming the difference in response to these treatment between normal and cancer cells.

To test whether cellular proliferative capacity may account for this difference, we measured expression of Sirt1 and TXNIP in embryonic stem cells known for rapid self renewal ability. The human embryonic stem cells,BG01V, were subjected to glucose restriction or treatment with either resveratrol or DHEA under similar conditions to those described in Figure 2. As shown in Figure 3B, both Sirt1 and TXNIP were found to be expressed in human embryonic stem cells; however, none of these genes was affected by resveratrol or DHEA. In contrast, limited glucose availability strongly inhibited TXNIP with no significant effect on Sirt1 expression, mirroring its action on cancer (Figure 2) and smooth muscle cells (Figure 3A). Taken together, these findings suggest that the pathways targeted by resveratrol and DHEA may be altered in cancer versus normal cells, and that the effects of these two treatment may be tissue specific. In contrast, the consistent inhibition of TXNIP by limited glucose availability in cancer cells as well as in differentiated smooth muscle cells and non-differentia-ted stem cells, is indicative of a potential role of this gene in mediating the anti-aging action of calorie restriction.

**Figure 4.**
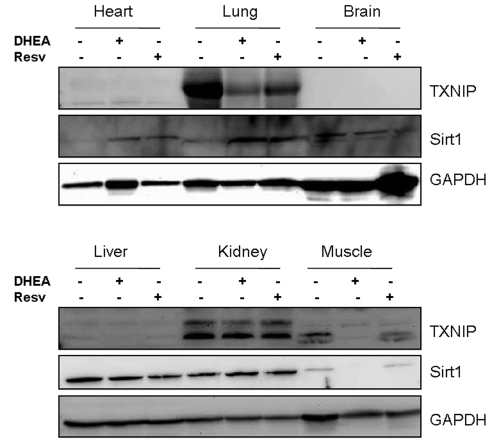
Tissue distribution of Sirt1 and TXNIP in mice and their regulation by resveratrol and DHEA. Mice CD1 strain were injected (*i.p*.) with 10 mg/Kg of either resveratrol or DHEA and after 2 days, the animals were sacrificed and organs harvested and processed by Western blot for the expression of Sirt1 and TXNIP. Staining with GAPDH was used as a loading control.

### Effect of resveratrol and DHEA on expression of Sirt1 and TXNIP in vivo

In order to determine whether the observed effects *in vitro* could be also valid *in vivo*, we have subjected the nude mice (CD1 strain, Charles River) to treatment with either resveratrol or DHEA (10 mg/Kg each) for 2 days. The animals were then sacrificed and major organs harvested to analyze expression of Sirt1 and TXNIP by Western blot.  As shown in Figure 4, the relative expression of these two genes was tissue specific. While Sirt1 is expressed in most organs tested; TXNIP was found to be expressed mainly in the lung, kidney and the muscle. Both resveratrol and DHEA inhibited the expression of TXNIP in the lung, however only DHEA had an inhibitory effect in the muscle. Sirt1 was not significantly affect in most tissues except in the muscle of animals treated with DHEA. Taken together, these findings suggest that resveratrol and DHEA are capable of regulating the expression of TXNIP and Sirt1, however in a tissue-specific manner.

**Figure 5.**
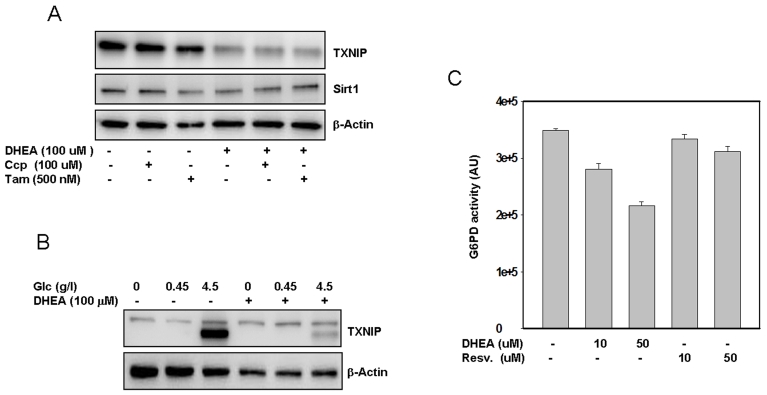
Putative mechanism(s) by which DHEA inhibits TXNIP. SaOS2 (panel **A**) were pre-incubated with Tamoxifen or CCP for one hour prior to addition of DHEA. After an additional incubation for 48 hours, proteins were extracted and probed by western blot for the expression of TXNIP and Sirt1. Panel **B**, The cells were treated with different concentrations of glucose with or without DHEA. After 48 hours in cultures, proteins were separated by electrophoresis and probed for TXNIP. Staining with β-actin in antibody was used as a loading control. Panel **C**, Effect of DHEA and resveratrol on G6PD activity. Protein extract (100 μg) was incubated with DHEA or resveratrol at the indicated concentrations, after 30 min at 37°C, the activity of G6PD was measured as described in the methods section. Data represent average of four determinations +/- SE.

### Putative mechanisms by which DHEA and resveratrol regulate TXNIP and Sirt1

The observation that TXNIP was consistently inhibited by all the treatments used in this study suggests that the signaling pathway leading to its expression may be a common target of these approaches. To define this pathway, we first investigated the implication of known targets of DHEA in mediating the effect of this hormone on TXNIP. Previous reports indicated that at the nuclear level, this hormone acts either through the peroxisome proliferator activated receptor alpha, pregnane X receptor, or the estrogen receptor [35]. At the plasma membrane, NMDA receptors and G-proteins were postulated to mediate the non-genomic action of DHEA [36,37]. In the cytoplasm, DHEA was found to act  as  a  non-competitive  inhibitor  of t he  glucose 6 phosphate dehydrogenase [38], a key glycolytic enzyme with a potential role in regulation of TXNIP. However, a putative role of these pathways in the regulation of TXNIP and/or of Sirt1 has not been described.

After subjecting SaOS2 cells to treatment with either an NMDA antagonist 3-[(+/-)-2-carboxypiperazin-4-yl] propyl-1-phosphonic acid (CPP), or an anti-estrogen drug (Tamoxifen), followed by cellular exposure to DHEA, the results (Figure 5A) indicate that neither CCP nor Tamoxifen reversed the inhibitory action of DHEA on TXNIP. Tamoxifen only partially reversed the effect of DHEA on Sirt1. Similar results were also obtained by using the RGC cells (Supplemental data 2), suggesting that these pathways may not play an important role in mediating the action of DHEA on TXNIP at least.

### Comparative effects of DHEA and resveratrol on activation of the glucose 6 phosphate dehydrogenase

Independently of the putative mechanisms described above, we have observed that cellular pretreatment with DHEA almost completely blocked the effects of glucose on TXNIP expression (Figure 5B), suggesting that this hormone may act, at least in part, by interfering with the glycolityc pathways that regulate TXNIP expression. Since DHEA has also been shown to inhibit the glucose 6 phosphate dehydrogenase, G6PD [38], we investigated whether this possibility may account for the observed effects of this hormone on TXNIP. The results presented in Figure 5C show that the activity of G6PD was indeed strongly inhibited and in a dose dependent manner by DHEA. Of note, since this *in vitro* assay is based on the production of NADPH (half of which is produced by G6PD and the rest by other enzymes such as Malic acid dehydrogenase and isocitrate dehydrogenase [39], the data (Figure 5C) suggest that  the inhibitory effect of DHEA  on G6PD could be much higher than the 50% observed. Resveratrol has no effect on G6PD (Figure 5C) suggesting that it may act through a different pathway to regulate TXNIP.

**Figure 6.**
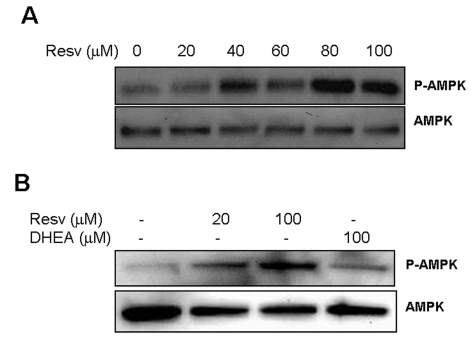
Effect of Resveratrol and DHEA on phosphorylation of AMPK. Panel **A**, SaOS2 cells were treated with increasing concentrations of resveratrol for 48 hours, after what, proteins were extracted and probed either with anti-phospho-AMPK (Thr172), or with antibody to AMPK. Panel **B**, RGC cells were incubated with 20 or 100 μM resveratrol or DHEA 100 μM for 48 hours, and then probed for phosphor-AMPK and AMPK as described above.

### Comparative effects of DHEA and resveratrol on AMP-activated protein kinase

**Figure 7.**
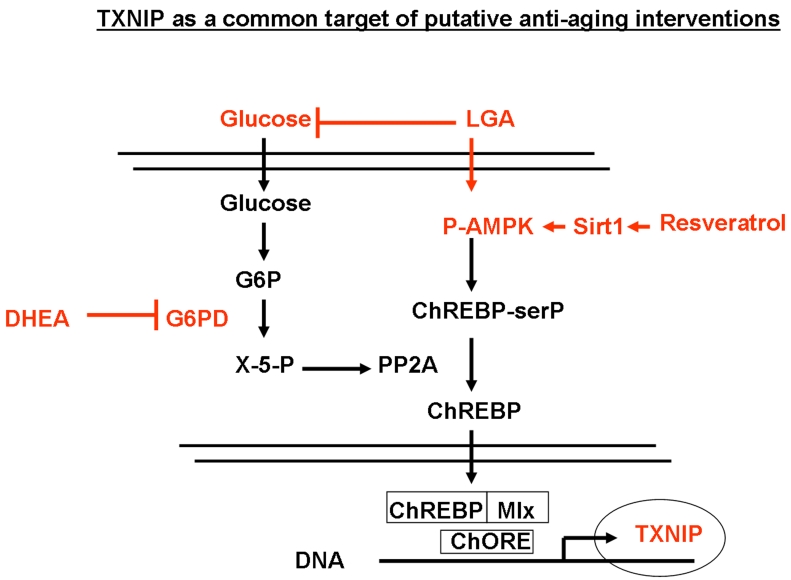
Regulation of TXNIP through the glycolitic pathway and its modulation by limited glucose availability (LGA), resveratrol and DHEA. Down regulation of glucose levels is known to increase the AMP/ATP ratio which in turn activates AMPK, leading to increased serine phosphorylation of ChREBP. Phosphorylated ChREBP is unable to translocate into the nucleus and form a functional complex with Mlx that is required for TXNIP expression. In addition, reduced glucose levels would inhibit, the pentose pathway and lead to the inhibition of the phosphatase PP2A. This will also result in the accumulation of phosphorylated ChREBP in the cytoplasm and further inhibition of TXNIP expression. DHEA acts on this glycolytic pathway mainly through the inhibition of G6PD activity. Resveratrol would act through the induction of Sirt1-mediated phosphorylation of AMPK, leading to enhanced phosphorylation ChREBP and inhibition of its nuclear translocation. This mechanism sheds light on TXNIP as a common downstream target for putative anti-aging interventions that affect metabolism.

Recent investigations of the mechanisms by which resveratrol improves metabolism revealed that it induces phosphorylation of the AMP-mediated protein kinase (AMPK) [40], an enzyme that phosphorylates and inactivates the carbohydrate response element binding protein ChREB. However, the relevance of this action to the regulation of TXNIP by resveratrol and the possible implication of DHEA in regulating this pathway are not yet investigated. The results presented in Figure 6A show that resveratrol readily activates this enzyme (p-AMPK) in the retinal glial cells (RGC) without affecting expression of the corresponding gene (AMPK). Resveratrol also induced AMPK phosphorylation in the osteosarcoma cells SaOS2 (Figure 6B), however DHEA was without effect. These findings, in addition to those presented in Figure 5D, suggest that, although both molecules inhibit TXNIP, DHEA and resveratrol act through separate branches of the glycolytic pathway mediated by G6PD and AMPK respectively. Also, in light of the recent finding that Sirt1 is able to activate AMPK [41], our data suggest that TXNIP may be regulated by Sirt1 through this pathway (Figure 7). Further confirmation of this link will help establish TXNIP inhibition as an important determinant for evaluating the efficacy of putative anti-aging interventions. The finding that limited glucose availability is the only approach that consistently inhibited expression of TXNIP in the all the systems used in this study, is in agreement with this concept and suggests that, regardless of Sirt1 levels, decreased expression of TXNIP may have beneficial effects for delaying the aging process.

## Discussion

The main purpose of this study was to direct attention to the fact that antagonistic pathways implicated in the aging process must be simultaneously targeted for optimal anti-aging effects. As a proof of principle for this concept, we have chosen to analyze the regulation of Sirt1 and TXNIP in response to proven and unproven approaches thought to affect aging. Due to the antagonistic roles of these two genes in mediating the pathways that regulate oxidative stress and metabolism, we hypothesized that approaches that induce Sirt1 and inhibit TXNIP expression would be the most desirable. By comparing the effect of limited glucose availability, resveratrol and DHEA in this system (Figure 2), only limited glucose availability led to the expected results (induction of Sirt1 and inhibition of TXNIP), providing further support for the already established notion that calorie restriction is so far the only proven approach that consistently increase life span. In contrast, we have found that depending on the dosage and the cell type used, resveratrol produced the unwanted effects of either inducing TXNIP or inhibiting the expression of its own target, Sirt1 (Figure 2C and 2D). Based on this, optimal beneficial effects of resveratrol against aging-associated diseases would necessitate proper understanding of how this compound induces TXNIP at low doses and inhibits its own target at higher ones, a knowledge that will then be useful for the development of strategies to avoid these unwanted effects. In this regards, we have shown that AMPK is phosphorylated at Thr172 by resveratrol (Figure 6), and  in light of previous findings that AMPK is activated by phosphorylation at Thr172 and inhibited by phosphorylation at 485/491 [42], one could speculate that resveratrol may induce phosphorylation at 485/491 at low concentrations and at Thr172 at higher ones. This, and the mechanism(s) by which resveratrol inhibits the expression of its own target Sirt1, warrants further investigations to determine the conditions under which this compound should  be administered without negatively influencing its own beneficial effects.

Concerning the action of DHEA, the finding that this hormone inhibited, in a dose dependent manner, the expression of both Sirt1 and TXNIP puts it in an unfavorable position regarding its claimed beneficial effects against aging. Our findings rather provide an explanation for the controversy surrounding the use of DHEA in hormone replacement therapy for older population. However, since this hormone has at least one beneficial effect which is the inhibition of TXNIP, understanding the mechanism by which it inhibits Sirt1 may be helpful in the design of strategies to prevent this unwanted effect, and open new avenues for its use as an anti-aging therapeutic. In this regard, our results point to the possibility that the estrogen receptor could be a potential mediator of the undesirable effects of DHEA on Sirt1 (Figure 5A), and suggest that a combination of DHEA with an estrogen receptor antagonist (i.e. Tamoxifen) may improve the anti-aging action of this hormone.

While the effects of limited glucose deprivation were consistently similar in cancer and normal cells, those of resveratrol and DHEA were surprisingly different. *In vitro* experiments indicated that resveratrol but not DHEA affected TXNIP expression in aortic SMCs (Figure 3A), however, none of these two treatments altered the expression of this gene in embryonic stem cells (Figure 3B). *In vivo* the effects of resveratrol and DHEA were rather tissue specific (Figure 4) as they were limited mainly to the lung and muscle among all the organs tested. By comparison with the findings made in Figure 2 using cancer cells, the latter results suggest that the signaling pathways that regulate expression of Sirt1 and TXNIP may be altered in cancer versus normal tissues, particularly in stem cells where only limited glucose availability was able to affect the expression of TXNIP.

The interesting finding that TXNIP was inhibited by all the treatments approaches tested in this study prompted us to investigate the underlying mechanism.  This led to the observation that both DHEA and resveratrol utilize the glycolytic pathway to inhibit TXNIP, however, they acted through two separate pathways mediated respectively by the inhibition of G6PDH (Figure 5) and the activation of AMPK (Figure 6). As shown in Figure 7, expression of TXNIP appeared to be regulated, at least in part, by Sirt1 through activation of the AMPK, which in turn signals for the inhibition of nuclear translocation of ChREB and its association with Mlx1 [43].  This, in light of the recent findings that Sirt1 enhances the function of FOXO1α[44] and that the latter inhibits expression of TXNIP [26], is indicative of a negative regulatory relationship between Sirt1 and TXNIP through this pathway. Based on this, and due to its position far downstream of the glycolytic pathway regulated by Sirt1, TXNIP may represent putative target to mimic the effects of caloric restriction in a Sirt1-dependend or even independent manner.

In broader terms, our findings provided evidence for the concept that the development of strategies to delay the aging process must take into account the presence of antagonistic pathways such as those mediated by Sirt1 and TXNIP on oxidative stress and metabolism. The observation that TXNIP acts as a downstream mediator of Sirt1 and a common target of various effectors of the glycolytic pathway, suggests that decreased expression of TXNIP alone may prove to be a reliable marker to evaluate and/or to predict the efficacy of putative anti-aging therapies.

## Materials and methods

The following drugs and reagents were obtained from the companies cited: DHEA; (+/-) CPP, Tamoxifen  (Sigma, St. Louis, MO); Antibody to acetylated p53  was obtained from upstate biotechnology (Lake placide NY); antibody to beta-actin from Sigma  (St. Louis, MO); secondary antibodies conjugated to horseradish peroxidase from BioRad (Hercules, CA); Enhanced chemiluminescence reagents (ECL) from Amersham (Arlington Heights, IL); Immobilon-P transfer membrane for western blots from Millipore (Bedford, MA).

Human monocytic cells (THP-1), osteosarcoma (SaOS2), breast carcinoma MCF-7 cells, the rat retinal ganglion cells (RGCs), aortic smooth muscle cells (SMCs) and the human embryonic stem cells BG01V1 were purchased from ATCC (Rockville MA). Dulbecco's Modified Eagle's Medium (DMEM), DMEM/F12 (1/1) medium, knockout serum and fetal bovine serum (FBS) were obtained from Invitrogen (Carlsbad, CA).  Cancer cell lines, HUVEC and SMC cells were maintained in their corresponding culture media and subjected to treatment with resveratrol or DHEA at concentrations ranging from 0 to 80 μM, or incubated in culture media with decreased glucose concentrations. After 48 hours of incubation, the cells were washed with PBS and re-suspended in 100 μl of lysis buffer (50 mM HEPES pH 7.4, 150 mM NaCl, 100 mM NaF, 1 mM MgCl2, 1.5 mM EGTA, 10% glycerol, 1% Triton X100, 1 μg/ml leupeptin, 1 mM phenyl-methyl-sulfonyl-fluoride). Equal amounts of proteins were analyzed by Western blot for expression of Sirt1 and TXNIP.

The human embryonic stem cells were cultivated in DMEM/F12 (1:1) supplemented with 40% knockout serum, 10 mM L-glutamine, and 1 mM non essential amino acids, and 10 μg/ml bFGF, and grown on mouse embryonic fibroblasts (MEFs) as feeders. Treatments with resveratrol, DHEA, or reduced glucose content were carried out as described above. After 48 hours of incubation, the cells were washed with PBS, treated with collagenase for 5 min at 37°C. The detached colonies were pelleted by centrifugation at 500xg for 5 min to separate them from floating MEFS. The pellets were re-suspended in lysis buffer as described above and proteins processed by Western blot, as described previously [45], to measure the expression of Sirt1 and TXNIP. Briefly, equal quantities of protein were separated by electrophoresis on a 12% SDS‑PAGE gel and transferred to Immobilon-P membranes.  Proteins of interest were identified by reaction with specific primary and secondary antibodies linked to horseradish peroxidase and detected by chemiluminescence [45].

PCR reaction. TotalRNA was isolated using Qiagen Rneasy mini kit (QiagenInc., Valencia, CA) as recommended by the supplier. Total RNAwas quantified by OD at 260 nm. Using equal amount of total RNA (200ng), stimulated under various conditions, mRNA wasprimed with random hexamers, and complementary DNA (cDNA) wassynthesized from mRNA by TaqMan reverse transcription withMultiScribe reverse transcriptase (PE Applied Biosystems, Foster,CT) according to the manufacturer's description. The finalcDNA product was used for subsequent cDNA amplification bypolymerase chain reaction.cDNA was amplified and quantitated by using SYBR Green PCR reagentsfrom PE Applied Biosystems according to the manufacturer'sinstructions. The cDNA for GAPDH was amplified by using a specific forwardprimer (5'-GAA GGT GAA GGT CGG AGT C-3') and a specific reverse primer(5'-GAA GAT GGT GAT GGG ATT TC-3'). The primers for TXNIP were: ctg gcg taa gct ttt caa gg (forward) agt gca caa agg gga aac ac (reverse). The primers for Sirt1 were: 5'-ATA GCA CAC AAA CAT CAT GCA-3' (forward) and 5'-TTT ATG CAT AAA ACA CCC AGC-3' (reverse). The PCRreaction mixture (final volume 25 μl) contained 5 μlof cDNA, 1 μl of 10 μM forward primer, 1 μlof 10 μM reverse primer, 2.5 μl of PCR 10x SYBRGreen PCR buffer, 3 μl of 25mM MgCl_2_, 2 μl of dNTPmix (2.5 mM dATP, 2.5 mM dCTP, 2.5 mM dGTP, and 5 mM dUTP),0.125 μl of AmpliTag Gold DNA polymerase (5 units/μlAmpliTag Gold DNA polymerase), and 10.125 μl of H_2_O. The reaction was amplified with iCycler iQ MulticolorReal Time PCR Detector (Bio-Rad) for 37 cycles with meltingat 94 °C for 30 s, an annealing at 58 °C for 30 s, and extension at 72 °C for 1 min in iCycler iQ PCR 96-wellplates (Bio-Rad).

Glucose 6 phosphate dehydrogenase activity. Enzyme activity assays were performed using the Vybrant. Assay Kit (V-23111, Molecular Probes, Eugene, OR) with minor changes to allow monitoring of cytosolic enzyme glucose 6-phosphate dehydrogenase in protein extracts. Briefly cells from a 75 cm^2 ^flask were lysed in (50 mM HEPES pH 7.4, 150 mM NaCl, 100 mM NaF, 1 mM MgCl_2_, 1.5 mM EGTA, 10% glycerol, 1% Triton X100, 1 μg/ml leupeptin, 1 mM phenyl-methyl-sulfonyl-fluoride) and diluted to a final concentration of 0.5 mg/mg in PBS.  Samples containing 50 μl of this solution were added to a 96 well plate and incubated in the absence or the presence of DHEA or resveratrol for 10 min at room temperature. The 2X reaction mixture containing the G6PD substrate resazurin was prepared as recommended by the manufacturer. The reaction was then started by addition of 50 μl of the reaction mixture to the protein samples, followed by incubation for 10 min at room temperature. The plate is then placed in a fluorescence microplate reader set up at the excitation 530 and emission 580 nm and fluorescence values recorded.

In vivo experiments *. *Mice were injected with DHEA or resveratrol (10 mg/Kg each) and after 48 hours, the animals were sacrificed and tissues harvested. To measure the levels of TXNIP and Sirt1, tissues were homogenized on ice in 300 μl of lysis buffer per gram of tissue. The lysates were then centrifuged at 1, 000xg for 5 min at 4°C and proteins in the supernatant quantified and processed for Western blot as described above.

## Supplementary data

Supplementary Figure 1 Effect of resveratrol and DHEA on expression of TXNIP and Sirt1 in the breast cancer cells MCF7. Panel **A**, the retinal ganglion cells RGC. Panel **B**, and the monocytic cell line THP-1 Panel **C**. Cells were treated with resveratrol or DHEA at the indicated concentrations for 48 hours, after what, proteins were extracted and probed by Western blot using specific antibodies to these two genes. β-actin was used as a loading control.

Supplementary Figure 2 Role of NMDA receptor in mediating the action of DHEA in RGC cells. The cells were pre-incubated with CCP for one hour prior to addition of DHEA. After an additional incubation for 48 hours, proteins were extracted and probed by western blot for the expression of TXNIP and Sirt1. β-actin was used as a loading control.

## References

[1] Vijg J, Suh Y (2005). Genetics of longevity and aging. Annu Rev Med.

[2] Knight JA (2000). The biochemistry of aging. Adv Clin Chem.

[3] Smith J (2002). Human Sir2 and the 'silencing' of p53 activity. Trends Cell Biol.

[4] Hasegawa K, Wakino S, Yoshioka K (2008). Sirt1 protects against oxidative stress-induced renal tubular cell apoptosis by the bidirectional regulation of catalase expression. Biochem Biophys Res Commun.

[5] Wang F, Nguyen M, Qin FX, Tong Q (2007). SIRT2 deacetylates FOXO3a in response to oxidative stress and caloric restriction. Aging Cell.

[6] Alcendor RR, Gao S, Zhai P (2007). Sirt1 regulates aging and resistance to oxidative stress in the heart. Circ Res.

[7] Baur JA, Pearson KJ, Price NL (2006). Resveratrol improves health and survival of mice on a high-calorie diet. Nature.

[8] Trapp J, Jung M (2006). The role of NAD+ dependent histone deacety-lases (sirtuins) in ageing. Curr Drug Targets.

[9] Milne JC, Lambert PD, Schenk S (2007). Small molecule activators of SIRT1 as therapeutics for the treatment of type 2 diabetes. Nature.

[10] Bordone L, Guarente L (2005). Calorie restriction, SIRT1 and metabolism: understanding longevity. Nat Rev Mol Cell Biol.

[11] Bordone L, Motta MC, Picard F (2006). Sirt1 regulates insulin secretion by repressing UCP2 in pancreatic beta cells. PLoS Biol.

[12] Moynihan KA, Grimm AA, Plueger MM (2005). Increased dosage of mammalian Sir2 in pancreatic beta cells enhances glucose-stimulated insulin secretion in mice. Cell Metab.

[13] Guarente L (2000). Sir2 links chromatin silencing, metabolism, and aging. Genes Dev.

[14] Yamawaki H, Haendeler J, Berk BC (2003). Thioredoxin: a key regulator of cardiovascular homeostasis. Circ Res.

[15] Yamawaki H, Berk BC (2005). Thioredoxin: a multifunctional antioxidant enzyme in kidney, heart and vessels. Curr Opin Nephrol Hypertens.

[16] Yoshioka J, Imahashi K, Gabel SA (2007). Targeted deletion of thioredoxin-interacting protein regulates cardiac dysfunction in response to pressure overload. Circ Res.

[17] Patwari P, Higgins LJ, Chutkow WA, Yoshioka J, Lee RT (2006). The interaction of thioredoxin with Txnip. Evidence for formation of a mixed disulfide by disulfide exchange. J Biol Chem.

[18] Chen J, Saxena G, Mungrue IN, Lusis AJ, Shalev A (2008). Thioredoxin-interacting protein: a critical link between glucose toxicity and beta-cell apoptosis. Diabetes.

[19] Chen J, Hui ST, Couto FM (2008). Thioredoxin-interacting protein deficiency induces Akt/Bcl-xL signaling and pancreatic beta-cell mass and protects against diabetes. Faseb J.

[20] Qi W, Chen X, Gilbert RE (2007). High glucose-induced thioredoxin-interacting protein in renal proximal tubule cells is independent of transforming growth factor-beta1. Am J Pathol.

[21] Stoltzman CA, Peterson CW, Breen KT, Muoio DM, Billin AN, Ayer DE (2008). Glucose sensing by MondoA:Mlx complexes: a role for hexokinases and direct regulation of thioredoxin-interacting protein expression. Proceedings of the National Academy of Sciences of the United States of America.

[22] Parikh H, Carlsson E, Chutkow WA (2007). TXNIP regulates peripheral glucose metabolism in humans. PLoS Med.

[23] Chutkow WA, Patwari P, Yoshioka J, Lee RT (2008). Thioredoxin-interacting protein (Txnip) is a critical regulator of hepatic glucose production. J Biol Chem.

[24] Chondrogianni N, de C M Simoes D, Franceschi C, Gonos ES (2004). Cloning of differentially expressed genes in skin fibroblasts from centenarians. Biogerontology.

[25] Yoshida T, Kondo N, Oka S (2006). Thioredoxin-binding protein-2 (TBP-2): its potential roles in the aging process. Biofactors.

[26] de Candia P, Blekhman R, Chabot AE, Oshlack A, Gilad Y (2008). A combination of genomic approaches reveals the role of FOXO1a in regulating an oxidative stress response pathway. PLoS ONE.

[27] Guarente L, Picard F (2005). Calorie restriction - the SIR2 connection. Cell.

[28] Lamberts SW, van den Beld AW, van der Lely AJ (1997). The endocrinology of aging. Science.

[29] Fukui M, Kitagawa Y, Ose H, Hasegawa G, Yoshikawa T, Nakamura N (2007). Role of endogenous androgen against insulin resistance and athero- sclerosis in men with type 2 diabetes. Curr Diabetes Rev.

[30] Gescuk BD, Davis JC Jr (2002). Novel therapeutic agents for systemic lupus erythematosus. Curr Opin Rheumatol.

[31] Hernandez-Morante JJ, Perez-de-Heredia F, Lujan JA, Zamora S, Garaulet M (2008). Role of DHEA-S on body fat distribution: gender- and depot-specific stimulation of adipose tissue lipolysis. Steroids.

[32] Farr SA, Banks WA, Uezu K, Gaskin FS, Morley JE (2004). DHEAS improves learning and memory in aged SAMP8 mice but not in diabetic mice. Life Sci.

[33] Perrini S, Natalicchio A, Laviola L (2004). Dehydroepiandrosterone stimulates glucose uptake in human and murine adipocytes by inducing GLUT1 and GLUT4 translocation to the plasma membrane. Diabetes.

[34] Gutierrez G, Mendoza C, Zapata E (2007). Dehydroepiandrosterone inhibits the TNF-alpha-induced inflammatory response in human umbilical vein endothelial cells. Atherosclerosis.

[35] Webb SJ, Geoghegan TE, Prough RA, Michael Miller KK (2006). The biological actions of dehydroepiandrosterone involves multiple receptors. Drug Metab Rev.

[36] Debonnel G, Bergeron R, de Montigny C (1996). Potentiation by dehydroepiandrosterone of the neuronal response to N-methyl-D-aspartate in the CA3 region of the rat dorsal hippocampus: an effect mediated via sigma receptors. J Endocrinol.

[37] Charalampopoulos I, Alexaki VI, Lazaridis I (2006). G protein-associated, specific membrane binding sites mediate the neuroprotective effect of dehydroepiandrosterone. Faseb J.

[38] Ziboh VA, Dreize MA, Hsia SL (1970). Inhibition of lipid synthesis and glucose-6-phosphate dehydrogenase in rat skin by dehydroepiandrosterone. J Lipid Res.

[39] Lawler JM, Demaree SR (2001). Relationship between NADP-specific isocitrate dehydrogenase and glutathione peroxidase in aging rat skeletal muscle. Mech Ageing Dev.

[40] Dasgupta B, Milbrandt J (2007). Resveratrol stimulates AMP kinase activity in neurons. Proceedings of the National Academy of Sciences of the United States of America.

[41] Lan F, Cacicedo JM, Ruderman N, Ido Y (2008). SIRT1 modulation of the acetylation status, cytosolic localization, and activity of LKB1. Possible role in AMP-activated protein kinase activation. J Biol Chem.

[42] Hurley RL, Barre LK, Wood SD (2006). Regulation of AMP-activated protein kinase by multisite phosphorylation in response to agents that elevate cellular cAMP. J Biol Chem.

[43] Minn AH, Hafele C, Shalev A (2005). Thioredoxin-interacting protein is stimulated by glucose through a carbohydrate response element and induces beta-cell apoptosis. Endocrinology.

[44] Nakae J, Cao Y, Daitoku H (2006). The LXXLL motif of murine forkhead transcription factor FoxO1 mediates Sirt1-dependent transcriptional activity. J Clin Invest.

[45] Rebbaa A, Chou PM, Mirkin BL (2001). Factors secreted by human neuroblastoma mediated doxorubicin resistance by activating STAT3 and inhibiting apoptosis. Mol Med.

