# Supplementary figures and images for: Dual targeting of the antagonistic pathways mediated by Sirt1 and TXNIP as a putative approach to enhance the efficacy of anti-aging interventions
Source: Aging (Albany NY). 2009 Mar 31;1(4):412–24. doi: 10.18632/aging.100035 (PMC2830051; doi:10.18632/aging.100035)

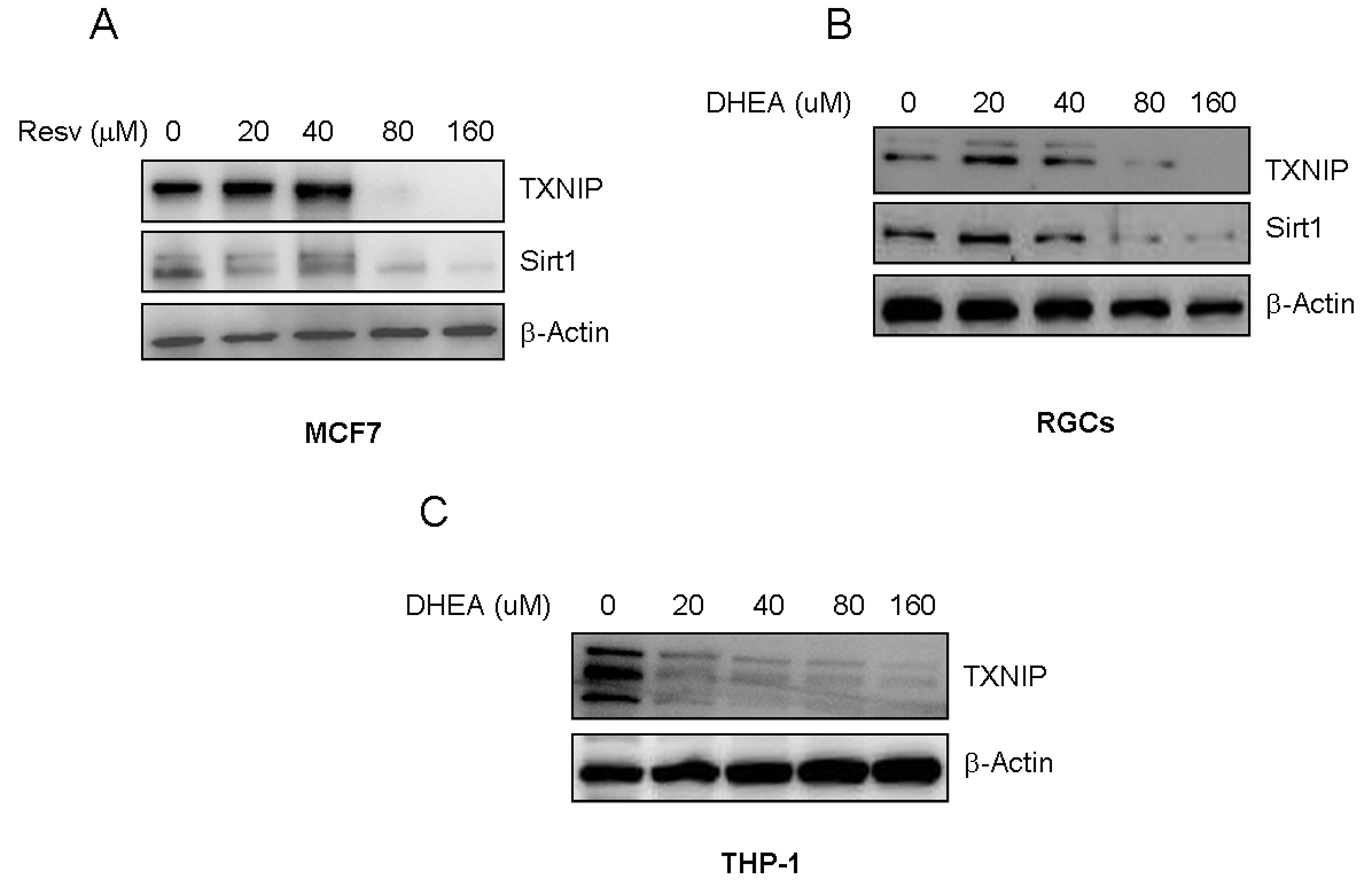

Supplement: Supplementary Figure 1 — Panel A, the retinal ganglion cells RGC. Panel B, and the monocytic cell line THP-1 Panel C. Cells were treated with resveratrol or DHEA at the indicated concentrations for 48 hours, after what, proteins were extracted and probed by Western blot using specific antibodies to these two genes. β-actin was used as a loading control. [file aging-01-412-s001.tif]

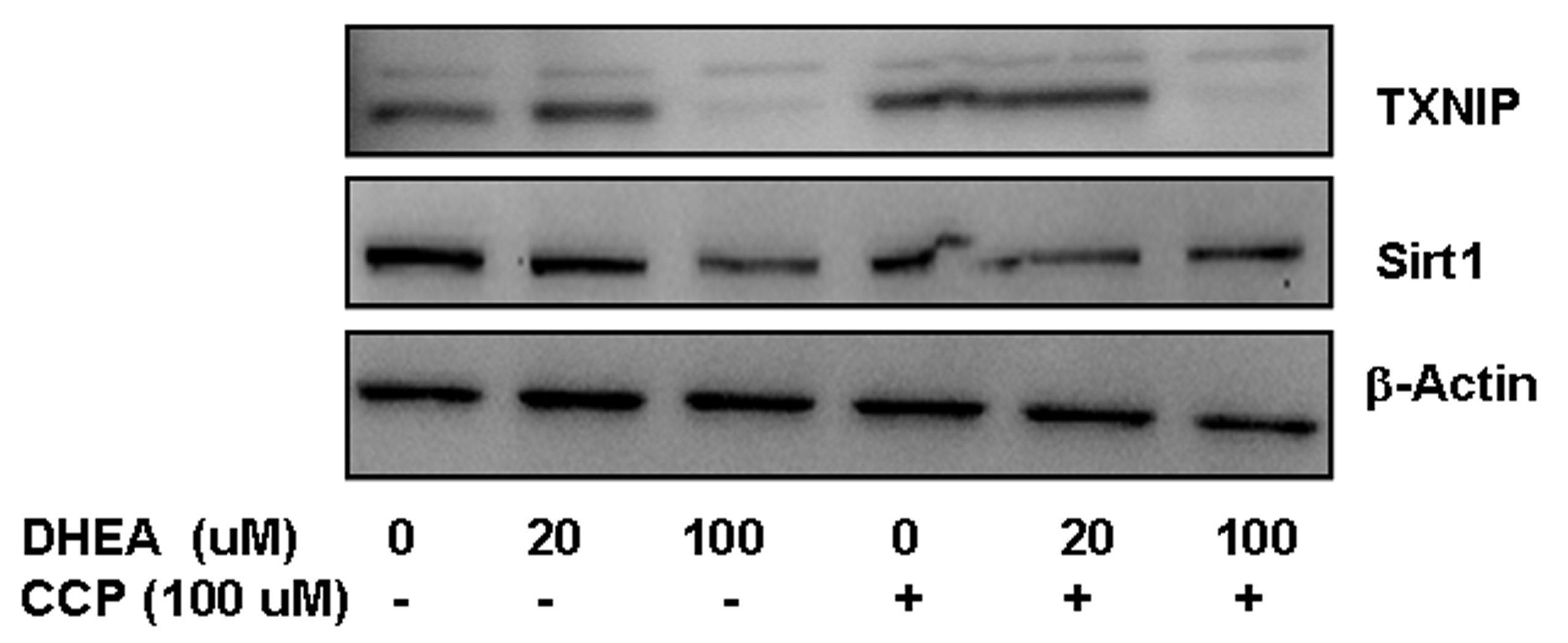

Supplement: Supplementary Figure 2 — The cells were pre-incubated with CCP for one hour prior to addition of DHEA. After an additional incubation for 48 hours, proteins were extracted and probed by western blot for the expression of TXNIP and Sirt1. β-actin was used as a loading control. [file aging-01-412-s002.tif]
